# Computational Simulation and Prediction on Electrical Conductivity of Oxide-Based Melts by Big Data Mining

**DOI:** 10.3390/ma12071059

**Published:** 2019-03-31

**Authors:** Ao Huang, Yanzhu Huo, Juan Yang, Guangqiang Li

**Affiliations:** 1 The State Key Laboratory of Refractories and Metallurgy, Wuhan University of Science and Technology, Wuhan 430081, China; huangao@wust.edu.cn (A.H.); 15527617291@163.com (Y.H.); liguangqiang@wust.edu.cn (G.L.); 2 National Engineering Research Center for E-learning, Central China Normal University, Wuhan 430079, China

**Keywords:** oxide melts, data mining, electrical conductivity, big data

## Abstract

Electrical conductivity is one of the most basic physical–chemical properties of oxide-based melts and plays an important role in the materials and metallurgical industries. Especially with the metallurgical melt, molten slag, existing research studies related to slag conductivity mainly used traditional experimental measurement approaches. Meanwhile, the idea of data-driven decision making has been widely used in many fields instead of expert experience. Therefore, this study proposed an innovative approach based on big data mining methods to investigate the computational simulation and prediction of electrical conductivity. Specific mechanisms are discussed to explain the findings of our proposed approach. Experimental results show slag conductivity can be predicted through constructing predictive models, and the Gradient Boosting Decision Tree (GBDT) model is the best prediction model with 90% accuracy and more than 88% sensitivity. The robustness result of the GBDT model demonstrates the reliability of prediction outcomes. It is concluded that the conductivity of slag systems is mainly affected by TiO_2_, FeO, SiO_2_, and CaO. TiO_2_ and FeO are positively correlated with conductivity, while SiO_2_ and CaO have negative correlations with conductivity.

## 1. Introduction

As an oxide-based melt, molten slag plays an important role in the materials and metallurgical industries. Electrical conductivity is one of the most basic physical–chemical properties of oxide-based melts and is of important significance because the metallurgical reactions such as electroslag remelting are based on the electrochemical principle, especially the conductivity of molten slag in the process of smelting. Different slag systems with complex compositions have significant influence on the optimization of metallurgical processes and reduction of energy consumption. It is known that the conductivity of slag is mainly affected by slag’s composition and temperature. Many research efforts have been invested in the investigation of the electrical conductivity of slags in order to obtain a comprehensive understanding of the electrochemical mechanism of the slag–metal reaction. It is easily found that existing research studies related to conductivity can be classified into two categories: traditional experimental measurement and automatic prediction based on data mining methods. 

For traditional conductivity investigation, researchers claimed that the conductive mechanism of molten slag is directional migration of the negative and positive ions under the effect of the electric field in the slag. The electric conductivity of the slag can be changed by the difference of conductive ions and the polymerization state of these ions. In order to avoid the change of slag composition under long-term direct current, high frequency alternating current is often utilized to reduce the measurement error in practical measurement. Ogino [1] proposed measuring the electrical conductivity of Electroslag Remelting (ESR) fluxes containing fluoride slag by using a four-electrodes method with alternating current. Some research [2,3] has shown that this method is one of the most commonly used methods to measure the electrical conductivity of slag. Alternating current (AC) impedance spectroscopy has also been widely used by researchers as it can effectively reduce the effects of electrode resistance and conductors [4]. Nevertheless, the above measuring methods are affected by various factors such as alternating current frequency, crucible materials, electrode materials, and so on in the actual measurement process, which certainly result in some measurement errors in the experimental process. Furthermore, these methods are time-consuming and labor-intensive, which can hinder the application of industrialization to some extent. 

With the rapid development of information technology, various types of data (such as: production systems, internet of things (IoT), environments) can be tracked and recorded, which results in exponential data growth. This is often called the ‘big data era’. Data-driven decision making, which refers to the practice of making decisions based on the analysis of data rather than purely on intuition [5], is considered the most significant value of the big data era. This idea has been widely applied in many fields, such as government management [6], economics [7], health care [8], education [9], as well as manufacturing [10]. Therefore, how to automatically analyze big data to support decision-making is vital. Because data mining as an interdisciplinary subfield of computer science aims to extract hidden patterns or knowledge from large data sets [11], many researchers started to apply data mining methods to discover meaningful information to support all levels of decision-making. Several innovative researchers first tried data mining to predict conductivity values in order to address the issues mentioned above. For example, the slag conductivity was quantitatively represented via a regression model based on the existing data about slag composition in the literature [12]. The authors reported that the conductivity was linearly correlated with the mole fraction of the basic oxides when it was assumed that CaO, MnO, and MgO existed in the form of Ca^2+^, Mn^2+^, and Mg^2+^, respectively. However, it is obvious that the number of examined samples was quite small, which limited the model’s generalizability. The good generalizability of a model in data mining means that this model can work well in other different environments. Zhang [13] used the Arrhenius formula to perform regression analysis on four slags including CaO–MgO–Al_2_O_3_–SiO_2_ based on the existing conductivity data. They obtained the optimized parameters of their regression model. However, this model cannot be work well in other slag systems because it is limited to specific temperatures and compositions. In summary, predictive models generated by these two conductivity studies have great limitations in terms of generalization due to a limited to regression method, small sample size, and specific conditions; therefore, these models cannot provide meaningful decision support for practical implementation.

The research objectives of this study are to collect a relatively big data set and to apply seven different data mining methods to build prediction models for automatically predicting conductivity values, obtaining a good generalization ability, and further providing decision-support for related stakeholders. Therefore, this study aimed to answer the following research questions: (1) In addition to the regression method, could other data mining methods be used to analyze and predict slag’s conductivity? (2) Which model is the best model for automatically predicting slag’s conductivity? (3) What is the minimum boundary of the collected data set for training a reliable prediction model? (4) How can we guide practitioners to improve conductivity in practice?

## 2. Materials and Methods

### 2.1. Data Collection and Preprocessing

Data for a total of 1796 slags (i.e., samples) were obtained by techniques such as AC four-probe and AC impedance spectroscopy. Data were collected from the SLAG ATLAS for 746 slags [14], and data for the other 1050 slags were found in references [2,3,4,15,16,17,18,19,20,21,22,23,24,25,26,27]. Fifteen common variables, including 14 variables on component proportions and one temperature variable, were collected as input variables as shown in Table 1. It was found that there were significantly different ranges among different component-proportion variables and the temperature variable in the dataset. If these raw input variables are directly used without any scaling, it results in two issues: (1) the convergence speed of the training model is very slow; and (2) the prediction performance is worsened. Therefore, it is essential to normalize all input variables to a range of 0–1.

Slag’s conductivity may be a numeric or nominal variable from the view of statistics when the temperature and slag composition are determined. In order to model slag’s conductivity performance, slag’s conductivity value needs to be transformed into a three-categorical variable as a prediction target, which labels all samples as high, medium, or low. With regard to high or low criterion, different practitioners have different guidelines. In this study, obtaining at least 10 (Ω·cm)^−1^ or greater than 10 (Ω·cm)^−1^ can be considered as high; slags with conductivity values less than 1 (Ω·cm)^−1^ are labelled as low; and the remaining conductivity values (i.e., 1 ≤ conductivity < 10) are medium. Therefore, after data cleaning, the dataset contained 7.68% high conductivity samples, 28.95% medium conductivity samples, and 63.37% low conductivity samples. Table 1 lists all original and derived variables for the following modelling and comparisons.

After data preprocessing and transforming, this analysis adopted K-fold cross validation to avoid overfitting. Because of the unbalanced data distribution, K was determined as five (K = 5) to maintain the model’s stability. The K-fold stratified method randomly split the dataset into five folds, which indicated that four folds were used for training and one was for validation.

### 2.2. Prediction Methods

**Figure 1:**
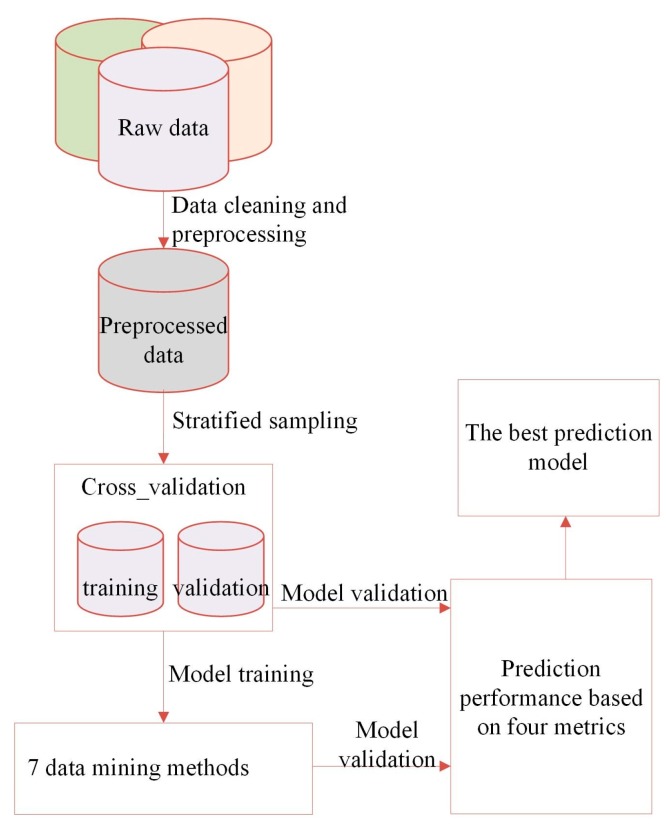
The logic flows for the selection of the best prediction model.

The whole data mining process for selecting the best prediction model is shown in Figure 1. Python environment was used for data modeling and analysis.

Seven different machine learning algorithms that are commonly used in data mining were selected to construct prediction models for predicting and identifying slag’s conductivity values. What follows are brief descriptions of the data mining methods used in this study.

- Logistic regression (LR) as a generalization of linear regression can be used for predicting binary or multiple-class target variables. Rather than perfecting a point estimate of the event itself, it calculates the probability of a categorical variable (e.g., good/medium/bad) based on a number of input variables [28].
- Decision Tree (DT) is a powerful classification algorithm based on partitioning. In each step, it partitions the data based on one variable until all data in each node have the same category label or all variables have been used. Different partitioning criterions (such as information entropy, information gain ratio, and Gini index) represent different algorithms, such as ID3 [29], C4.5 [30] and Classification and Regression Trees (CART) [31]. In this study we employed CART Decision Tree for modeling.
- Naïve Bayes (NB) applies Bayes’ theorem to calculate a conditional probability distribution over the output of a function to achieve classification. Obviously, this algorithm has an assumption of independence among the predicting variables [32].
- Support Vector machine (SVM) aims to find a hyperplane to optimally separate different categories of data. It assumes that the larger the margin between these parallel hyperplanes, the better the generalization error of the classifier will be [33]. Therefore, finding the maximum-margin hyperplanes from both categories is an optimization problem. In this study, linear kernel function was adopted.
- Artificial Neural Network (ANN) tries to mimic the brain structure to model extremely complex non-linear relationships [34]. It has one input layer, multiple hidden layers, and one output layer. Neurons in the upper layer are connected to neurons in the next layer with different weights. In this study, we used two hidden layers.
- K-Nearest Neighbor (KNN) is different from the algorithms described above as it does not require training of the parameters. Based on a majority vote of its K neighbors, KNN classifies a sample [35]. Therefore, calculating the distance between the samples to select its K nearest neighbors is crucial. In this study, we used the default K value (K = 5) to calculate five nearest neighbors to classify the sample.
- Gradient Boosting Decision Tree (GBDT) belongs to a family of ensemble models. The general idea of boosting trees is to generate a number of simple trees, where each tree is built based on the prediction residuals of the preceding tree [36]. Due to learning from the previous tree, the misclassification can be minimized. On the basis of the traditional boosting tree, the GBDT algorithm employs a gradient descent algorithm to speed up the convergence.

For all of these machine learning algorithms, the same data were used to train, validate, and compare with each other. 

Measuring the overall prediction accuracy is commonly used in data mining. However, due to the three categorical and imbalanced characteristics of our dataset (the percentage of samples with high and medium conductivity is less than 40%), it is crucial to correctly identify the minority categories (medium and high). As the study aims to identify medium and high conductivity samples, obtaining a prediction accuracy that is as high as possible for these two categories is vital. Meanwhile, when improving the prediction accuracy of the minority categories, the benefits of the majority category should also be considered. Therefore, another metric, geometric mean (GM), is selected to represent a trade-off in multiple categorical and imbalanced datasets. 

On the other hand, the confusion matrix [37], also described as the error matrix, can be used to intuitively calculate the above indicators to evaluate the performance of prediction models for classification tasks in the field of data mining. In our classification task, the confusion matrix is represented in Table 2. Next, we introduce the meaning of this confusion matrix.

- For the high category: TP_2_ (True Positive) means a slag whose conductivity value is no less than 10 (Ω·cm)^−1^ and the model also correctly predicts the slag as high; FP_2_j_ (j = 1 or N) means a slag conductivity is less than 10 (Ω·cm)^−1^, but the model predicts the slag’s conductivity as high.
- For the medium category: TP_1_ (True Positive) means a slag whose conductivity value is between 1 (Ω·cm)^−1^ and 10 (Ω·cm)^−1^, and the model also correctly predicts the slag as medium; FP_1_j_ (j = 2 or N) means a slag’s conductivity does not belong to the medium category, but the model misclassifies it as medium.
- For the low category: TN (True Negative) denotes a slag whose conductivity value is actually less than 1 (Ω·cm)^−1^ and the model also correctly predicts the slag as low; FN_i_ (i = 1 or 2) denotes a slag with conductivity greater than 1 (Ω·cm)^−1^, but the model incorrectly predicts the slag’s conductivity as low.

Therefore, based on the description of the confusion matrix of the three categorical classifications, these indicators can be computed through the following equations [37].

(1) $$\mathrm{Accuracy}=\frac{TP_{1}+TP_{2}+TN}{TP_{1}+TP_{2}+TN+FP_{2\_1}+FP_{2\_N}+FP_{1\_2}+FP_{1\_N}+FN_{2}+FN_{1}}$$

(2) $${\mathrm{Sensitivity}}_{1}=\frac{TP_{1}}{TP_{1}+FP_{2\_1}+FN_{1}}$$

(3) $${\mathrm{Sensitivity}}_{2}=\frac{TP_{2}}{TP_{2}+FP_{1\_2}+FN_{2}}$$

(4) $$\mathrm{Specificity}=\frac{TN}{FP_{2\_N}+FP_{1\_N}+TN}$$

(5) $$GM=\sqrt{{\mathrm{Sensitivity}}_{1}*{\mathrm{Sensitivity}}_{1}*Specificity}$$

In this study, both accuracy and GM value are selected to evaluate the models’ overall performances. Finally, all prediction models were optimized by the five-fold cross validation to avoid potential overfitting.

### 2.3. Model Robustness

Two common issues (i.e., “underfitting” and “overfitting” [38]) of machine learning algorithms may result from multiple reasons, including inappropriate training set size, a lot of noisy data, too few features, and so on. Under the limitation of data collection, we just focused on the range of training size to investigate each model’s robustness in this study. Each modeling method has a training size range in which prediction models can perform accurately. Too small a training size may not provide enough information to construct prediction models with satisfactory accuracy, namely “underfitting”. Too large a training size may result in learning too many detailed relationships that only exist in the training dataset so that the prediction model cannot fit well on the validation (i.e., future new data), namely “overfitting”. Therefore, selecting an appropriate training size to make the prediction model work accurately is very important, especially for the high conductivity category.

Different subsets of the training dataset were used to train the models, and then the models’ performances were evaluated based on the GM value for the purpose of identifying the minimum and maximum training size boundaries of the models. If the sample size was not in the range, the prediction models developed in this study could not accurately predict the samples’ conductivity.

In order to create training sets with different sizes, the four-folds data (about 1436 samples) as a training dataset were split into 30 clusters of 47 or 48 samples based on the stratified sampling to keep the high/medium/low ratios similar or the same. After splitting, based on the overall 7.68% high and 28.95% medium conductivity ratios, each cluster had 3 high conductivity samples and 13 medium slag samples. This was the lowest number of samples in each category (high or medium or low) that was required for training models. The number of clusters was increased from one to 30 in the training phase to investigate the model’s performance under different training sizes, which indicated the range of training size was 47–1436 samples. For each training size, the clusters were selected randomly five times and the average results were reported.

### 2.4. The Identification of Significant Factors

A general consensus in the data mining field is that improving prediction accuracy for a target task and identifying significant predictive factors have the same priority. The former can inform researchers and practitioners about which slag has high conductivity, but the researchers and practitioners still do not know how to effectively optimize the components or conditions to improve conductivity unless they have been informed about which factors can significantly affect the slag’s conductivity.

In general, there are two ways to find the most important factors that significantly influence slag’s conductivity value. Correlation analysis is a common method to examine relationships between the predicted variable (i.e., high/medium/low conductivity) and the input variables. The larger the correlation coefficients, the more important the variable. Obviously, the important factors obtained from the correlation analysis are independent of the prediction models. Another way is to employ a surrogate modelling method (i.e. using another model to explain a complex model (“Surrogate model,” n.d.) [39]) to interpret results generated from the best prediction model. The surrogate models aim to simulate results from the best models. Therefore, for our analyses, we kept the same sets of input variables, but the target variables were replaced with the predicted values by the best model. The decision tree algorithm was able to simulate the best model with 100% accuracy. This gave us a rough understanding of the important factors based on the correlation analysis, then we carefully checked the significant factors based on the surrogate model. 

## 3. Results

### 3.1. Selecting the Best Model for Predicting Conductivity

This experiment aimed to answer the following research questions: (1) Can data mining methods be used to identify and predict slags’ conductivity? (2) By comparing the prediction performance of seven prediction models on the validation dataset, which model outperforms others in terms of identifying three category samples (i.e., having the highest accuracy and GM value)?

**Figure 2:**
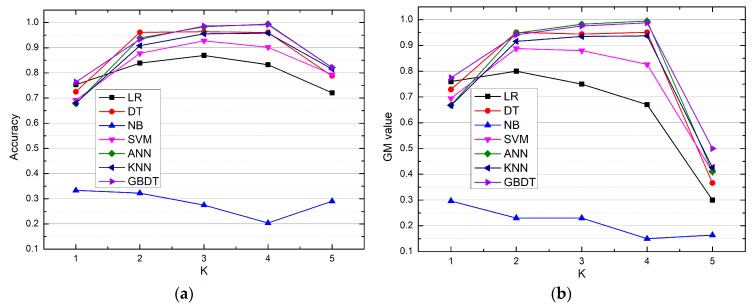
Five-fold validation results of different prediction models. (**a**) Accuracy, (**b**) geometric mean (GM) values.

As stated earlier, overall accuracy and GM value were used to evaluate the models’ performances. Figure 2 presents the five-fold validation results (accuracy and GM value) of every prediction model.

Figure 2 shows that the NB model performed more poorly than the other models. The NB model had the lowest overall accuracy and GM value, which means this model misclassified a great number of conductivity samples. The averages of the five-fold cross validation on the validation dataset were further computed and are listed in Table 3.

Both Figure 2 and Table 3 all indicate that the LR model also had relatively poor prediction performance, especially for the medium category. The remaining five prediction models (DT, SVM, ANN, KNN, and GBDT) obtained an overall prediction accuracy of over 80% and achieved relatively high GM values. These five prediction models seem to be more suitable for identifying and predicting a sample’s conductivity than the NB and LR models. Furthermore, Table 3 shows that the GBDT model performed significantly better than the other models in terms of overall accuracy and GM value. In addition, considering that GBDT as an ensemble model is more robust than models based on a single algorithm, it can be concluded that the GBDT model is the best model for predicting slag’s conductivity with 90% overall accuracy and more than 88% sensitivity.

Therefore, this experiment showed that data mining methods can not only be used for modeling slag’s conductivity, but also to achieve satisfactory prediction performance. The GBDT model is more suitable than other models for automatically predicting slag’s conductivity.

### 3.2. Investigating the Robustness of the Best Prediction Model

The first experiment identified that the model based on the GBDT method was the best prediction model with relatively satisfactory performance. Therefore, the robustness of the best prediction model to the size of the training dataset was further investigated. A limited dataset size cannot provide enough information, so the prediction accuracy is low. A training dataset with a very large size may result in overfitting, so that the prediction performance of the generated model decreases when used on new data.

**Figure 3:**
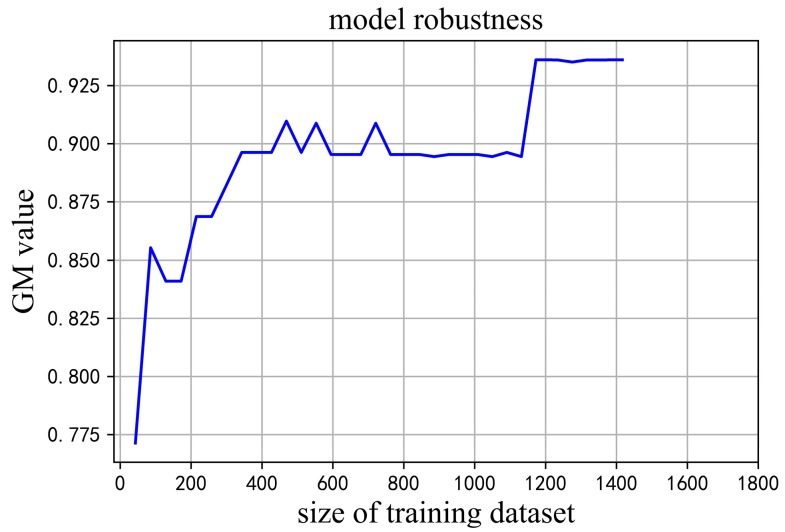
GM values of the models based on different training sizes.

The number of samples was increased from 47 to 1436 in the training phase and the evaluation results are show in Figure 3. After the size of the training dataset was 1188, increasing the training size could not further improve the prediction performance. Increasing the size of the training dataset yielded the same results as the 1188 sample. This means that containing at least 1188 samples in the training dataset is necessary to ensure the reliability of the prediction model.

### 3.3. Finding the Most Significant Factors

Correlation analysis between input variables and the target variable was conducted to get a rough understanding of the significant factors. Generally, only variables with correlation coefficients of more than 0.3 are considered as important factors that have strong relationships with the target variable. Therefore, only four factors (SiO_2_, TiO_2_, CaO, and FeO), which had −0.59, 0.58, −0.57, and 0.31 correlation values, respectively, were initially important factors. The remaining 11 factors had weak correlation relationships with the target variable.

**Figure 4:**
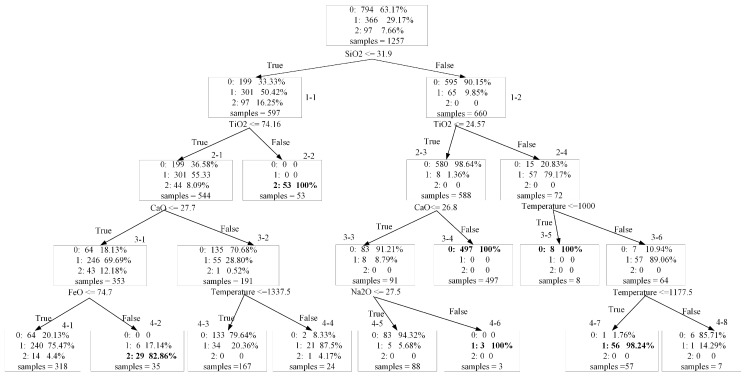
The surrogate model results of the GBDT prediction model.

Then, another set of experiments that adopted the surrogate modelling method to interpret results generated from the best prediction model (i.e., GBDT model) were conducted to reveal significant factors. Because GBDT is an ensemble model (a combination of many decision trees), the surrogate model is very complex as well. Due to aim of finding the most significant division variables, only the top five layers of the surrogate model are shown in Figure 4. 

Figure 4 shows that the order of factor importance is SiO_2_, TiO_2_, CaO, and FeO. “0” denotes the low conductivity slags whose conductivity values are less than 1 (Ω·cm)^−1^, “1” means the slags with medium conductivity values, while “2” means high conductivity slags. To enhance readability, paths for identifying medium or high conductivity slag samples are marked with an asterisk.

*Rule 1-1: SiO_2_ ≤ 31.9% (0/1/2:0.33/0.5/0.17)
- Rule 1-2: SiO_2_ > 31.9% (0/1/2:0.9/0.1/0)
- Rule 2-1: 1-1 + TiO_2_ ≤ 74.16% (0/1/2:0.37/0.55/0.08)
*Rule 2-2: 1-1 + TiO_2_ > 74.16% (0/1/2:0/0/1)
- Rule 2-3: 1-2 + TiO_2_ ≤ 24.57% (0/1/2:0.99/0.01/0)
*Rule 2-4: 1-2 + TiO_2_ > 24.57% (0/1/2:0.21/0.79/0)
*Rule 3-1: 1-1 + 2-1 + CaO ≤ 27.7% (0/1/2:0.18/0.7/0.12)
- Rule 3-2: 1-1 + 2-1 + CaO > 27.7% (0/1/2:0.71/0.29/0)
- Rule 3-3: 1-2 + 2-3 + CaO ≤ 26.8% (0/1/2:0.91/0.09/0)
- Rule 3-4: 1-2 + 2-3 + CaO > 26.8% (0/1/2:1/0/0)
- Rule 3-5: 1-2 + 2-4 + Temperature ≤ 1000 °C (0/1/2:1/0/0)
*Rule 3-6: 1-2 + 2-4 + Temperature > 1000 °C (0/1/2:0.11/0.89/0)
*Rule 4-1: 1-1 + 2-1 + 3-1 + FeO ≤ 74.7% (0/1/2:0.2/0.75/0.05)
*Rule 4-2: 1-1 + 2-1 + 3-1 + FeO > 74.7% (0/1/2:0/0.17/0.83)
*Rule 4-3: 1-1 + 2-1 + 3-2 + Temperature ≤ 1337.5 °C (0/1/2:0.71/0.29/0)
*Rule 4-4: 1-1 + 2-1 + 3-2 + Temperature > 1337.5 °C (0/1/2:0.08/0.87/0.05)
- Rule 4-5: 1-2 + 2-3 + 3-3 + Na_2_O ≤ 27.5% (0/1/2:0.94/0.06/0)
*Rule 4-6: 1-2 + 2-3 + 3-3 + Na_2_O > 27.5% (0/1/2:0/1/0)
*Rule 4-7: 1-2 + 2-4 + 3-6 + Temperature ≤ 1177.5 °C (0/1/2:0.02/0.98/0)
- Rule 4-8: 1-2 + 2-4 + 3-6 + Temperature > 1177.5 °C (0/1/2:0.86/0.14/0)

Firstly, there are two paths that can lead to a higher chance of the slag being identified as high (i.e., conductivity > 10 (Ω·cm)^−1^), including (rules 1-1 and 2-2) as well as (rules 1-1 and 2-1 and 3-1 and 4-2). For example, rule 1-1 denotes if slag’s SiO_2_ component is less than 31.9%, the high probability is only 0.17. When Rule 1-1 was satisfied and the slag’s TiO_2_ component was greater than 74.16%, the high probability increased from 0.17 to 1. This path indicates that the higher the component of TiO_2_, the higher the conductivity. Another path shows that if slag components contained a small amount of SiO_2_, TiO_2_, and CaO, and contained a large amount (>74.7%) of FeO, the high probability was 0.83. In summary, containing higher TiO_2_ or FeO component is beneficial for greatly improving slag’s conductivity to a very high level.

Secondly, four paths that can result in a higher chance of the slag being identified as medium (i.e., 1 ≤ conductivity < 10 (Ω·cm)^−1^), are identified in Figure 4, including (rules 1-1 and 2-1 and 3-1 and 4-1), (rules 1-1 and 2-1 and 3-2 and 4-4), (rules 1-2 and 2-3 and 3-3 and 4-6), and (rules 1-2 and 2-4 and 3-6 and 4-7). For example, rule 1-1 denotes if slag’s SiO_2_ component was less than 31.9%, the medium probabilities increased to 0.5. Similarly, when Rule 1-1 and 2-1 and 3-1 were satisfied and slag’s FeO component was less than 74.7%, the medium probability increased from 0.29 to 0.75; when Rule 1-1 and 2-1 and 3-1 were satisfied and slag’s temperature was over 1337.5, the medium probability increased from 0.29 to 0.87. Rule 1-2 denotes if slag’s SiO_2_ component was more than 31.9%, the medium probability was only 0.1. However, when Rule 1-2 and 2-3 and 3-3 were satisfied and slag’s Na_2_O component was greater than 27.5%, the medium probability increased to 1. The last path means if slag’s components met (SiO_2_ > 31.9% and TiO_2_ > 24.57%) and slag’s temperature was between 1000 and 1177.5, the medium probability increased from 0.1 to 0.98. These four paths reveal that if slag contains SiO_2_, TiO_2_, CaO, FeO, and Na_2_O components and has an appropriate temperature condition, it will easily to have a relatively high conductivity value based on the above paths for optimizing and adjusting guidance. 

The remaining factors that were not listed in Figure 4 (such as: MgO, Fe_2_O_3_, K_2_O, etc.) had little effect on slag’s conductivity. Therefore, based on the combination of correlation analysis results and surrogate model results, it is concluded that the most important factors related to slag’s conductivity are SiO_2_, TiO_2_, CaO, and FeO, followed by temperature and Na_2_O. Researchers and practitioners need to pay more attention to these significant factors to improve slag’s conductivity. In addition, paths generated by the surrogate model as shown in Figure 4 can be utilized as practical guidance for decision-making. 

## 4. Discussion

The aims of this study were to explore whether data mining methods can be used to analyze and predict slag’s conductivity, which model performs best in predicting medium or high conductivity values, whether the identified best prediction model can provide reliable prediction outcomes, and to further investigate the significant factors for providing meaningful insights to related stakeholders to support their decision-making process. 

### 4.1. The Importance of High Sensitivity and Gm Values 

In the comparison of prediction performances of the seven prediction modeling methods, the best one for capturing high conductivity samples was the Naïve Bayes (NB) with 93% sensitivity, but this model also misclassified a great number of medium or low conductivity samples as wrong categories. The overall reliability of the NB prediction model was only 28%, which indicates the NB model cannot be implemented in practice.

Obtaining high sensitivity and GM values is more meaningful than high overall accuracy in an imbalanced dataset [40]. For our conductivity dataset (high conductivity and medium conductivity ratios were 7.68% and 28.95%, respectively), if a prediction model just simply classified any sample as the majority category, it also achieved 63.37% overall accuracy. Obviously, this model does not make any sense for the minority category. Meanwhile, for multiple category classification tasks (like our conductivity dataset), making a trade-off among categories is also important [41]. Therefore, the GM values of the remaining six prediction models were compared, and it was found that GBDT was the best prediction model. It is not surprising because GBDT is a combination of many decision trees, so it will naturally have lower prediction errors in predicting new data than any of the individual models that it comprises (i.e., the DT model) [42]. In addition to the advantage of high prediction performance, the GBDT model as an ensemble model is more robust to noisy data than other models.

### 4.2. The Size Range for Training a Reliable Prediction Model for Conductivity

This study also investigated the range of training dataset size in which the prediction model can work accurately. In this study, the prediction performance of the GBDT model reached its best performance when the training dataset contained at least 1188 samples. The ratio of high conductivity samples was less than 10% in the whole training dataset, which means a subset of 1188 samples had less than 118 samples with high conductivity values. Therefore, we can infer that the low performance of the model based on the training dataset with smaller than 1188 samples was due to the limited number of high conductivity samples. However, the prediction performance remained stable from 1188 samples to 1436 samples, which was the full training dataset. Thus, the minimum boundary for training prediction models is 1188 samples, which avoids underfitting. 

When the size of the training dataset is very large, overfitting may occur. In the case of overfitting, the model remembers irrelevant details of the training data, which may prevent it from finding the underlying relationships and results in a poor generalization [43]. In this study, the maximum boundary was unclear, but is certainly larger than 1436 samples, and needs to be investigated in the future via collecting more data.

### 4.3. The Significant Factors for Predicting Conductivity

The conductivity of slag is usually caused by electron flow and ion migration. The oxide states are different under high temperature conditions, which result in a big difference in the conductivity value. Due to the limitation of data collection, the conductivity values in this study were affected by the temperature, the types of oxide, and slag content. The specific mechanism of TiO_2_, FeO, SiO_2_, and CaO on the conductivity are analyzed in the following paragraphs.

Experimental results show that the correlation between conductivity and TiO_2_ can reach +0.58, and the positive correlation is more obvious with the increase of temperature. The conductivity of the slag usually varies with the valence state of titanium ions. When Ti^3+^ exists in the slag, it usually exhibits high electrical conductivity, because the electrostatic field of Ti^3+^ is smaller than Ti^4+^, and Ti^3+^ has stronger mobility. Besides, according to the study of Tranell et al. [44], the low-valent titanium ion (Ti^3+^) has better stability at high temperature, so the low-valent titanium ion content further increases. In addition, the migration rate of ions under high temperature is faster, so it is not surprising that slags under this condition show high electrical conductivity characteristic.

Based on the results of the surrogate model, the addition of FeO increases the conductivity of the slag system. In iron-containing slag, if the trend of the covalent binding of ferrous ions and oxygen is strong enough, there will be a high covalent ion FeO_4_^5−^, rather than Fe^3+^ [22]. However, FeO_4_^5−^ has slower ion mobility and less contribution to charge transfer, so the main carrier of charge in molten slag is Fe^2+^. This means that the ionic conductivity of slag will increase with increasing FeO content. As a metal oxide with variable valence, FeO exhibits both electrical conductivity and ionic conductivity. According to the diffusion-assisted charge transfer model proposed by Barati and Coley [45], the charge transfer between divalent and trivalent iron ions can be regarded as a bimolecular reaction. When the total iron content is fixed, the conductivity is directly proportional to γ × (1 − γ), (γ is the ratio of ferrous ion to total iron ion). Thus, as the ferrous ions increase monotonically, the conductivity should first increase first and then decrease. In addition, there is a maximum conductivity when Fe^2+^/Fe^3+^ = 1. In summary, the ionic conductivity and electronic conductivity of the iron-containing slag will increase with increasing FeO content, and the electronic conductivity has the maximum value at Fe^2+^/Fe^3+^ = 1.

SiO_2_ is often negatively correlated with conductivity, and the correlation is −0.59. According to the ionization theory of slag, SiO_2_ is an acidic oxide in the molten slag. It can combine with O^2−^ to form anionic clusters such as SiO_4_^4−^ and Si_2_O_7_^6−^ in the melting process. Moreover, this large size polymerization anion migrates slowly under the action of an electric field, so the conductivity of silicate slag is poor. However, a slag system containing SiO_2_ can easily form low melting point compounds when coexisting with some oxides (such as Al_2_O_3_, CaO). Therefore, the electrical conductivity will be improved within a certain range.

In addition, there was a negative correlation between CaO and slag conductivity, and the correlation was −0.57. However, CaO is usually decomposed into Ca^2+^ and O^2−^ during the melting process to achieve electrical migration. Meanwhile, CaO can also form low melting point compounds with other oxides, which results in a high conductivity of the slag system. However, in our study, the slag data that contained CaO showed low conductivity characteristics. In order to further explore why CaO reduces the conductivity of the slag system, we analyzed the data of low-conductivity containing CaO, and found that these data mainly have the following characteristics:

- The content of CaO is less than 50%;
- The temperature range is mainly concentrated at 1300–1500 °C;
- The original composition of the slag system is mainly composed of Al_2_O_3_ and SiO_2_ in addition to CaO.

**Figure 5:**
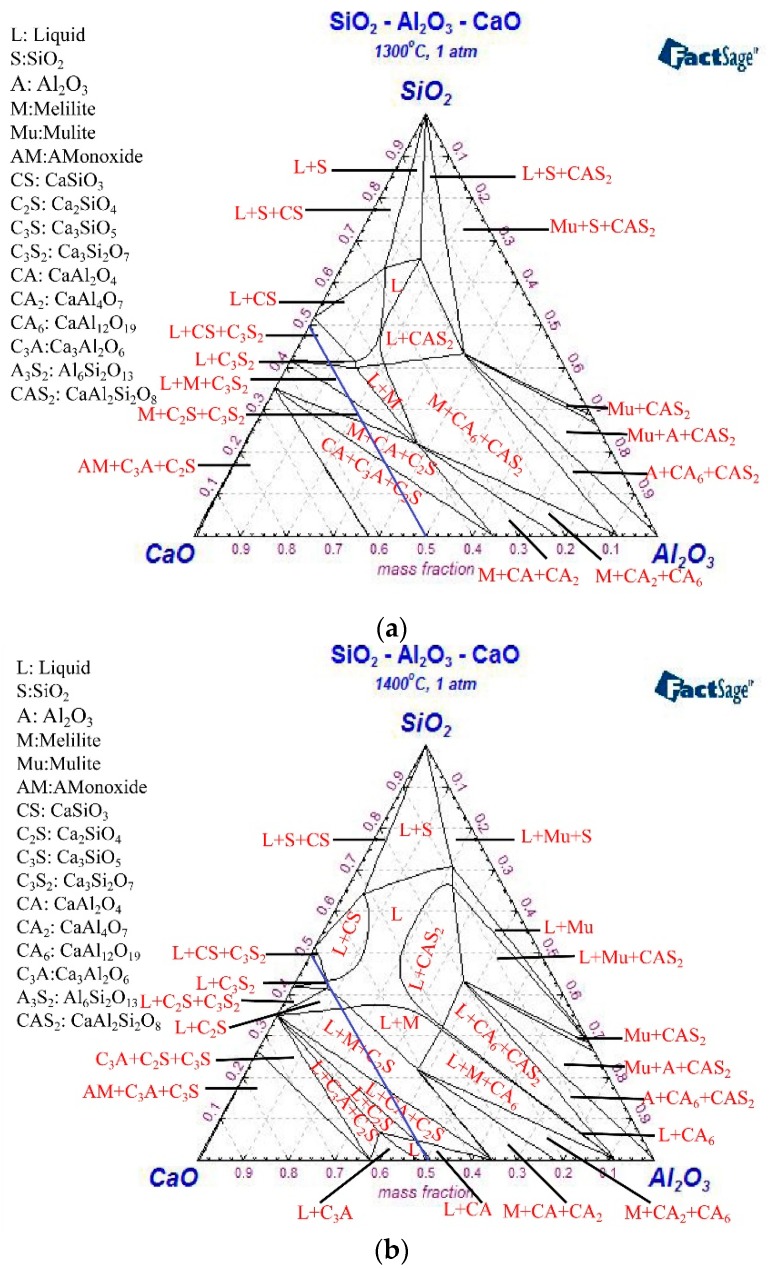

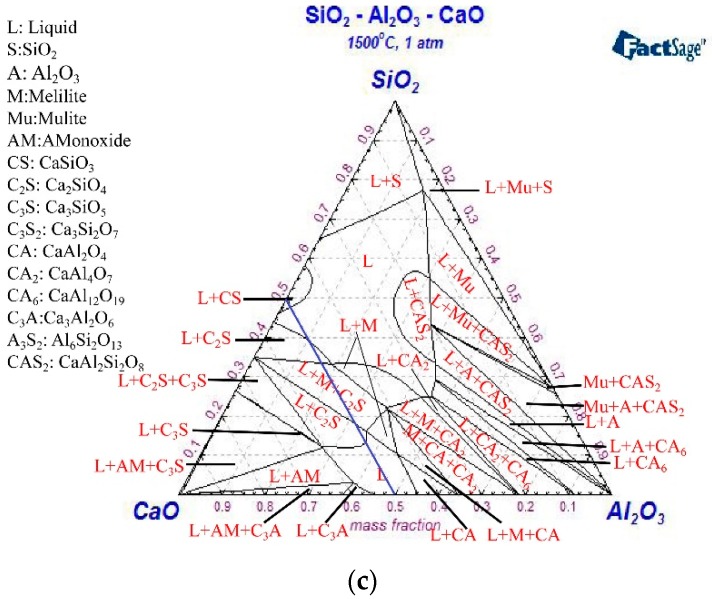
Phase diagram of the CaO–Al_2_O_3_–SiO_2_ system at temperatures of (**a**) 1300 °C; (**b**) 1400 °C; and (**c**) 1500 °C.

According to the above analysis results, Phase Diagram of FactSage 6.4 (Thermfact/CRCT, Montreal, QC, Canada and GTT-Technologies, Aachen, Germany) was used to calculate the isothermal phase diagram of the Al_2_O_3_–SiO_2_–CaO system [46]. FactPS and FToxid databases were selected, and the temperature was set to 1300 °C, 1400 °C, and 1500 °C. Figure 5 shows the calculation results of the phase diagram.

Figure 5 indicates that when the melt with CaO content was more than 50%, the majority of compounds in the melt were silicate. The structure of silicate was affected by Ca^2+^ concentration, consequently, the network structure of silicate was destroyed and the degree of polymerization of the melt decreased with the increase of CaO content. Meanwhile, with the increase of Ca^2+^ concentration, the conductivity of the melt was improved. While the CaO content was less than 50%, aluminate was dominant in the melt. With the increase of CaO content, Al^3+^ was compensated by Ca^2+^ so that it existed in the form of AlO_4_^5−^ and the degree of polymerization of the melt increased, which led to the decrease of the melt conductivity. In addition, due to the limitation of Ca^2+^ content, no redundant Ca^2+^ contributed to the increase of the melt conductivity. Therefore, the movement of ions was influenced so that the melt showed a low conductivity.

## 5. Conclusions

The results of this exploratory study indicate that data mining methods can be used for constructing prediction models to automatically predict electrical conductivity values of slags, which is convenient for guiding related research and industrial application compared with traditional experiments. Among the examined seven data mining methods, GBDT was is the best model for predicting slag’s conductivity with 95% overall accuracy and more than 89% sensitivity. In addition, the best prediction model’s robustness was also examined to demonstrate the reliability of prediction outcomes. Finally, based on the analysis results of the correlation analysis and surrogate model, it was also found that the slag’s conductivity was mainly affected by four factors, including TiO_2_, FeO, SiO_2_, and CaO. TiO_2_ and FeO were positively correlated with conductivity, while SiO_2_ and CaO had negative correlations with conductivity of the slag. It was revealed that CaO can lower the slag conductivity with the change of CaO content, which is different from the literature reports with small data testing.

## Figures and Tables

**Table 1:** Variables for data analysis.

Variables	Attribute	Description	Extent
FeO	Numeric	FeO mass fraction in slag	0–100%
Al_2_O_3_	Numeric	Al_2_O_3_ mass fraction in slag	0–100%
CaF_2_	Numeric	CaF_2_ mass fraction in slag	0–100%
Fe_2_O_3_	Numeric	Fe_2_O_3_ mass fraction in slag	0–100%
SiO_2_	Numeric	SiO_2_ mass fraction in slag	0–100%
CaO	Numeric	CaO mass fraction in slag	0–100%
MgO	Numeric	MgO mass fraction in slag	0–100%
SrO_2_	Numeric	SrO_2_ mass fraction in slag	0–44%
TiO_2_	Numeric	TiO_2_ mass fraction in slag	0–90%
K_2_O	Numeric	K_2_O mass fraction in slag	0–45%
MnO	Numeric	MnO mass fraction in slag	0–74%
Na_2_O	Numeric	Na_2_O mass fraction in slag	0–50%
ZrO_2_	Numeric	ZrO_2_ mass fraction in slag	0–24%
Cr_2_O_3_	Numeric	Cr_2_O_3_ mass fraction in slag	0–10%
Temperature	Numeric	Temperature of slag	300–2800 °C
Conductivity	Categorical	Slag’s conductivity value	0.00037–335.3 (Ω·cm)^−1^

**Table 2:** Confusion matrix for the three-categorical classification.

Total Samples		Predicted Category		
		High (P_2_)	Medium (P_1_)	Low (N)
Actual category	High (P_2_)	TP_2_	FP_1_2_	FN_2_
	Medium (P_1_)	FP_2_1_	TP_1_	FN_1_
	Low (N)	FP_2_N_	FP_1_N_	TN

**Table 3:** The averages of five-fold cross validation for seven prediction models.

Methods	LR	DT	NB	SVM	ANN	KNN	GBDT
Accuracy	0.80	0.88	0.28	0.84	0.88	0.86	0.90
Sensitivity_1_	0.61	0.82	0.43	0.77	0.90	0.84	0.88
Sensitivity_2_	0.85	0.85	0.93	0.86	0.84	0.84	0.89
Specificity	0.89	0.91	0.14	0.87	0.88	0.88	0.91
GM	0.66	0.79	0.21	0.74	0.80	0.78	0.84
